# Editorial: Scale Development and Score Validation

**DOI:** 10.3389/fpsyg.2020.00799

**Published:** 2020-04-22

**Authors:** Laura Badenes-Ribera, N. Clayton Silver, Elisa Pedroli

**Affiliations:** ^1^Department of Behavioral Sciences Methodology, University of Valencia, Valencia, Spain; ^2^Department of Psychology, University of Nevada, Las Vegas, NV, United States; ^3^Centro Neuropsicologia, Istituto Auxologico Italiano (IRCCS), Milan, Italy

**Keywords:** psychological testing, psychometrics, quantitative measurement, questionnaire, scale, reliability, validation

Scale development and validation of scores is not a job to be taken on lightly. Development is a rigorous process which is based on item generation and content validation using expert feedback and pre-testing. In fact, it may take numerous iterations for the scale to be economically feasible and yet convey the appropriate construct.

After the scale has been qualitatively developed, it goes through a rigorous quantitative examination to evaluate its score reliability and validation. This validation may include construct, concurrent, predictive, concurrent, and discriminant. For example, there are numerous techniques for evaluating construct validity such as using exploratory factor analysis (EFA) followed by confirmatory factor analysis (CFA) or using a structural equation model (SEM). Of course, determining the number of factors in an EFA can be quite a problem. Many researchers use the classic Scree test or Kaiser's eigenvalue-greater-than-1.0 technique. However, some studies suggest that these may not be the best techniques (e.g., Lloret-Segura et al., 2014). Other procedures have been developed that allegedly have better psychometric properties, such as Velicer's MAP, parallel analysis, Ruscio and Roche's CD technique, and Achim's NEST method.

Another problem with validation is that the participants are often a single sample (usually college students), which can limit the generalizability of the findings even though cross-validation could still be used. However, we are beginning to witness questionnaires or scales translated into a variety of languages so that factor structures and factor scores become comparable. This cross-cultural work may aid in assessing measurement invariance.

This Research Topic welcomed all types of empirical articles focused on the analysis of the psychometric properties of the measurement instruments in any psychological or social science area. A total of 107 authors contributed 22 articles to the Topic. These articles can be organized intro four issues: (1) Scale development with solid psychometric score validation techniques; (2) Cultural adaptation of developed scales (3) Validation of scores on developed scales, and (4) Invariance measurement of developed scales.

## Scale Development With Solid Psychometric Score Validation Techniques

Gorostiaga et al. developed and examined the psychometric properties of the Entrepreneurial Orientation Scale (EOS) in a sample of undergraduate students. The EOS showed good psychometric properties and its dimensions demonstrated concurrent relationships with self-efficacy and personal initiative. The EOS may be used to measure entrepreneurial orientation in the educational context and to evaluate interventions designed to promote an entrepreneurial spirit in schools, colleges, and universities.

Shek et al. developed and examined the psychometric properties of the Short form Service Leadership Behavior Scale (SLB-SF-38). This scale was based on the Service Leadership Model proposed by Po Chung. Both EFA and CFA were involved in the validation study. The SLB-SF-38 showed excellent internal consistency, concurrent validity, and factorial validity based on multigroup invariance analyses. The SLB-SF-38 may be used to measure service leadership behavior in the education, research, and personnel training contexts.

Wang D. et al. developed and examined the psychometric properties of a new instrument for depression under the framework of Cognitive Diagnosis Models (CDMs), referred to as CDMs-D. The CDMs-D, which showed good reliability and validity, measures all ten symptom criteria for depression defined in ICD-10 (World Health Organization, 2010) and covers five domains of depression defined by Gibbons et al. (2012). It can also provide both overall information on the severity of depressive disorders and assessment information on specific symptoms defined in the ICD-10, which could be useful for diagnostic and interventional purposes.

Wang J. et al. constructed and validated an instrument to measure psychological security in the area of urban residents' lives known as the Urban Residents Psychological Security Scale (URPS), which showed good reliability and validity using EFA and CFA. This scale can be used as an effective measurement tool for urban residents' psychological security and could be useful for better understanding of residents' demands and monitoring the implementation effects of policies.

Wingenbach et al. created and validated the Verbal Emotion Vignettes as stimulus set to elicit emotions (anger, disgust, fear, sadness, happiness, gratitude, guilt, and neutral) in Portuguese, English, and German. Hierarchical cluster analyses showed that the vignettes mapped clearly on their target emotion categories in all three languages. The final stimulus sets each include 4 vignettes per emotion category plus 1 additional vignette per emotion category, which can be used for task familiarization procedures in research. The high agreement rates on the experienced emotion in combination with the medium-to-large intensity ratings in all three languages suggest that the stimulus sets are suitable for application in emotion research (e.g., emotion recognition or emotion elicitation).

Zhang et al. developed and examined the psychometric properties of the Short-Form Inventory of Callous-Unemotional Traits (ICU, Essau et al., 2006, Chinese version of the ICU: Wang et al., 2017), which was designed to evaluate multiple facets of Callous-Unemotional traits in youths. The short form of the ICU with two factors and 11 items had the best model fit ICU in a Chinese male juvenile offender sample. Both the total and two factor scores showed acceptable internal consistence and convergent validity. The ICU-11 is a promising tool for assessing CU traits in the Chinese male detained juvenile sample.

## Cultural Adaptation of Developed Scales

Rizzo et al. developed the Italian version of the Existential Quest Scale (EQ) and examined factorial structure, internal consistency, discriminant validity, and measurement invariance across gender and age groups. CFA showed that the original one-factor structure was replicated, except for one-item that was removed from the subsequent analyses. Both the internal consistency of the eight-item scale as assessed by Cronbach's and discriminant validity were in line with those of the original study. Furthermore, they found evidence of full measurement invariance across gender and partial measurement invariance across age. Overall, the Italian version of the EQ is a promising tool for assessing flexibility on existential issues.

Ronzón-Tirado et al. adapted the Modified Version of the Conflict Tactics Scale [M-CTS (Neidig, 1986); Spanish adaptation: (Muñoz-Rivas et al., 2007)], in Mexican adolescents using an analysis of the linguistic and cultural variables, followed by a CFA, and the evaluation of Construct and Known Groups Validities. They culturally modified six items and verified the four-factorial structure of the questionnaire. The cultural adaptation of the M-CTS offered adequate reliability and validity scores and expanded the possibilities of comparing the prevalence of the problem between nations with a reliable instrument based on the same theoretical and methodological perspectives.

Yan et al. developed and examined the psychometric properties of the Chinese version of the Brief version of the Situational Test of Emotional Understanding (STEU-B) and the Brief version of the Situational Test of Emotional Understanding (STEM-B) (Allen et al., 2014, 2015) using the Item Response Theory method and criterion validity. The Chinese versions of the STEU-B and STEM-B scales showed psychometrically adequate measurements. These scales might be useful to capture employees' emotional understanding and emotional regulation as an alternative to ability tests of Emotional Intelligence.

## Validation of Scores on Developed Scales

Angel et al. examined the psychometric properties of the Enriched Life Scale (ELS, Team Red White Blue, 2017) developed to systematically capture and quantify the experiences of military veterans transitioning to civilian life. They used CFA to validate the factorial structure of the ELS in veterans and provided evidence of internal consistence, discriminant, and convergent validity. The ELS could be used in conjunction with diagnostic instruments that capture strain-related transition challenges (to include mental health disorders) to capture post-military service well-being.

Fung et al. assessed the dimensionality and psychometric properties of the Brief Self-Control Scale (BSCS, Chinese version Unger et al., 2016) in a sample of undergraduates using EFA and CFA. A shortened version of the 11-item BSCS with a four-factor structure had better psychometric properties and a good model fit in the CFA. This scale provides a comprehensive and handy measure for broader research in the context of mainland China or the Chinese diaspora.

Tindall and Curtis evaluated the factorial structure of the Need Satisfaction and Frustration Scale (NSFS; Longo et al., 2016) and its predictive validity in a sample of undergraduate students and individuals from the wider community using an SEM. They provided support to Longo et al. (2016, 2018), who stated that need frustration and need satisfaction are distinct constructs, and also gave further insight into the relationship between basic Need Frustration and common types of psychological health problems.

Willmer et al. examined psychometric properties of the 9-item Utrecht work engagement scale (UWES-9, Schaufeli et al., 2006) in a multi-occupational female sample using EFA and CFA. The EFA seemed to mainly favor a one-factor solution, which was shown to explain over 70% of the variance, but none of three different (one-, two-, and three-factor) models showed an overall good fit in CFA. Further research is needed to disentangle the possible effects of gender, nationality, and occupation on work engagement.

Xiao et al. examined the association between student-level information and communication technology (ICT) impact factors (the availability, use and attitudes toward ICT) and reading proficiency among early adolescents using a multiple linear regression model. They found that the students' ICT-related attitudinal factors concerning their interest in ICT and perceived autonomy in using it, rather than its availability and use, were closely associated with high reading proficiency.

## Analyzing the Measurement Invariance of Developed Scales

Dagnall et al. evaluated the scale's factorial structure of the Belief in Science Scale (BISS), which assesses the degree to which science is valued as a source of superior knowledge using parallel analysis, EFA, CFA, and invariance testing across gender. They found support to invariance of form, factor structure, and item intercepts for a one-factor model. The scale showed good internal consistency and one-factor solution, signifying that this was consistent with the single-factor model advocated by Farias et al. (2013).

Frey-Clark et al. determined that scores on the Statistical Anxiety Scale (SAS, O'Bryant, 2017) manifest in the same way for students in online and traditional statistics courses using a measurement invariance test.

Martí-Vilar et al. examined the invariance of the Prosocial Behavior Scale (PS, Caprara et al., 2005) across gender and country and psychometric properties in three Hispanic countries (Argentina, Spain, and Peru) using SEM methodology. They also evaluated reliability and internal consistency at both score and item level.

Meng et al. evaluated the factorial structure of the 10-item Connor-Davidson Resilience Scale (CD-RISC-10) in the Chinese elders using CFA and the measurement invariance across gender using multigroup CFA. They found that a single-factor model fitted CD-RISC-10 data well, both for the total sample and for each gender group. Factorial invariance across genders was also supported.

Vagos et al. evaluated the factorial structure of the Morningness-Eveningness-Stability-Scale (MESSi) using CFA and measurement invariance across gender and age using multigroup CFA. They found a three-factor structure for the MESSi and full measurement invariance of the three-factor model for gender and age.

Zhao et al. determined the factor structure of the 15-item Geriatric Depression Scale (GDS-15) in a sample of Chinese elders using CFA and the measurement invariance across gender using multigroup CFA. They found that a three-factor model best fits the structure of the GDS-15, and that measurement invariance across gender was supported, fully assuming different degrees of invariance.

On the other hand, recent developments in statistics have provided new analytical tools for assessing the validity of the scales. French et al. conducted a simulation study to examine the performance of the Generalized Mantel-Haenszel (GMH) procedure and a Multilevel GMH (MGMH) procedure for the detection of uniform differential item functioning (DIF) in the presence of multilevel data with polytomous items. They found differences in DIF detection when the analytic strategy matches the data structure. The GMH had an in?ated Type I error rate across conditions and thus an artificially high power rate, and the MGMH had good power rates while maintaining control of the Type I error rate. Finally, Hayduk et al. detailed the relevant procedural steps to conduct a fusion validity and illustrated the procedure using the Leadership scale from the Alberta Context Tool (ACT) with care aides working in Canadian long-term care homes.

This Research Topic includes different examples of scale development and validation protocols, each one with rigor and scientific peculiarity. We had analyzed four different aspects of this wide field of knowledge: scale development with solid psychometric score validation techniques, cultural adaptation of developed scales, validation of scores on developed scales, and invariance measurement of developed scales. It's important to show how variegate these processes could be with the aim of promote the use of different scientific-based techniques.

## Author Contributions

LB-R, EP, and NS all helped in writing the editorial.

## Conflict of Interest

The authors declare that the research was conducted in the absence of any commercial or financial relationships that could be construed as a potential conflict of interest.
